# The Qualifications Necessary for the Dental Practitioner

**Published:** 1896-08

**Authors:** P. T. Smith

**Affiliations:** Denver, Col.


### THE QUALIFICATIONS NECESSARY FOR THE DENTAL PRACTITIONER.[1]

BY DR. P. T. SMITH, DENVER, COL.

So much has been said and written upon this subject under various headings that it might seem well-nigh useless, or perhaps tiresome, to listen to the result of an effort to formulate any new ideas that may direct our thoughts over a subject seemingly so thoroughly analyzed, but rejoicing over the fact that good has been developed by the energy thus expended, and recognizing that much yet remains to be done, it is fitting that we recall to our minds the history of the past for the benefit of the future. The limited vantage ground occupied to-day sufficiently widens the horizon of our future duties and demands, to determine the course we must follow to place the profession on a footing of undoubted usefulness. The cause of the narrowed sphere of dentistry is readily recognized and easily defined, and should lead us to an honest acknowledgment of the peculiarities of the situation, and stimulate us to assiduous efforts towards an improved condition.

It is not a polymathic subject with a question as to which is right. The true position is easily demonstrable logically and practically, leaving no doubt as to the proper course for the profession to follow.

It has been argued by a respectable faction that dentistry is not necessarily a part of the medical profession *per se*, but an independent and distinct body with title and functions bearing distant collateral relations. This unfortunate and unwarranted conclusion greatly increases the unjust subordination of the dental curative department, and gives force to the sentiment of our limited professional curriculum.

Let us see our duty as unselfish eyes may easily do, fighting down mercenary and sordid ambition that saps the growth of the purer form of life.

The dental student should only be taken from the ranks of the medical profession, commencing the study of the specialty where the completion of the necessary foundation for the medical diploma left off.

All branches of the parent profession derive support and development, and necessarily produce fruitage, for evil and good in

---

[1] Read before the Colorado State Dental Association, June 11, 1896.

proportion to their dependence and helpful support from the main trunk. A complete medical knowledge for the dentist creates a power of conception important and necessary in diagnostication of preliminary inharmony which effect otherwise conditions of the peripheral tissues that fall particularly under their care.

Almost every duty devolving upon the dental practitioner needs for its best accomplishment the support and guidance of a complete medical knowledge. It is a necessary mental discipline for the fullest analysis of the merest and most commonplace function he is called upon to discharge. No two individuals represent the same physical condition, and he may be without sufficient skill to exploit a true diagnosis, such as can most easily and surely be done by aid of a full medical understanding.

There could be much realized by the compulsory attendance at our dental offices if the watchful proprietor at these offices was universally a medical graduate, and had taken this degree, as he always should, prior to his passing the threshold of the dental department. Our dental colleges are pursuing an entirely wrong course in the method of their teaching. The result is a lame, unfinished, unsupporting, misleading delusion, which strands the hopeful, well-wishing, conscientious student on the reefs that beset the path of their future course through life. But a small per cent. of the practising dentists have had the ambition to voluntarily endow themselves with the necessary mental pabulum so needed and obtained in a medical course.

The medical profession recognizes this lack of thorough education necessary for the demands made upon us. They realize that all and nothing short of a complete course which bears upon every specific point must be shown in our curriculum before we can be eligible to our full duties and their recognition.

For the fullest and most honorable fruitage of our calling, a great change must be adopted in our process of selecting material and preparing new dentists for the great future welfare of the people,—the dignity and fellowship of the profession.

There should be but one profession, and that the medical, from which many radiating specialists can diverge with their respective distinctive titles which shall be adjunctive. As the fountain cannot rise higher than its source, we must expect to receive a credit only commensurate with the amount of education represented in our present limited course, and while these pseudo-dental colleges are permitted to exist and dole out half mental rations, filling up the country to the four winds with sophisticated swaddlings, we may cover our faces with shame and sink to the common level of the uneducated wage-earner, and accept the pittance deposited upon our professional sacrificial altar. Then the shoemaker, the barber, and the dentist may walk hand in hand, financially and socially, with no hope beyond the realm of abiding ignorance.

When dental colleges all through the land are denominated respectable by a national college faculty association, and will turn out dentists with twenty-eight to fifty didactic lectures in the course of three years on some of the most important subjects, and the disinterested medical professors teaching these same dental students will pass them, at their final examination, on a thirty to a thirty-five per cent. examination, then our hopes may really sink below the evening clouds of despair. There is no attempt at technics applicable to the course prior to selecting or training students; no careful or trained clinics. The people have a right to demand true professional representation.

The mechanical department must be entirely sequestrated from the specialty. It now serves to clog and hinders the true advancement of the profession.

I notice at the colleges, and usually with the incipient practitioner, that the prosthetic, so-called, is regarded as the acme of their common ambition. And even with this, I think I may truthfully say, I never saw but one man who knew prosthetic dentistry, and who was able to place it in a technological aspect.

So great is the unwarranted and sordid ambition of all men, especially Americans, to coin every effort and faculty of our existence into money, counting the measure of our success wholly by the volume of its accumulation in our possession, that no elevation of our specialty above this murky position is possible until we have unshackled our fettered souls, and learn to adore the shrine of a more noble purpose which shall uplift us far above the grovelling level of mist and darkness.

I blame the colleges for much of our benighted condition. There are ten times more of the poor, sickly excuses in existence than is demanded. They forge their mercenary course along by solicitation, advertising, and begging throughout the country for students, for the sole purpose of embellishing the names of some people with an unwarranted prefix and the amount of money which can be realized from the method.

